# Miliary Tuberculosis With Gastrointestinal Involvement in a Young Undocumented Immigrant: A Case Report

**DOI:** 10.7759/cureus.105325

**Published:** 2026-03-16

**Authors:** Mohammed Khaleefah, David Parvizi, Karrar Khaleefah, Farheen Basith, Khushman K. Bhullar, Marco Valladres, Kim Nguyen, Mohammed Siddiqui

**Affiliations:** 1 Family Medicine, Chino Valley Medical Center, Chino, USA

**Keywords:** adult gastroenterology, infectious disease pathology, miliary tuberculosis, multiplle organ failure, reticulonodular opacities

## Abstract

Miliary tuberculosis is a severe form of disseminated *Mycobacterium tuberculosis* infection resulting from hematogenous spread of the organism. It is characterized by numerous small nodular lesions throughout the lungs and other organs and can involve multiple systems, including the gastrointestinal tract. The condition is associated with high morbidity and mortality, particularly when diagnosis and treatment are delayed.

This case report describes a 31-year-old male who presented to Chino Valley Medical Center on November 11, 2025, with a five-month history of progressive weight loss, intractable vomiting, fatigue, and chronic productive cough. On admission, he was severely cachectic and reported an inability to tolerate food for over two weeks. Imaging and laboratory evaluation revealed disseminated tuberculosis with a miliary pattern on chest imaging and profound malnutrition. CT imaging of the abdomen without contrast revealed multiple dilated proximal small bowel loops with a transition point in the distal ileum, consistent with small bowel obstruction. Sputum cultures later grew extended-spectrum beta-lactamase (ESBL)-producing *Escherichia coli*. While this organism is an uncommon cause of primary pneumonia, the finding raised concern for secondary infection or possible aspiration in the setting of severe illness and prolonged hospitalization. Anti-tuberculosis therapy with RIPE (rifampin, isoniazid, pyrazinamide, and ethambutol) was initiated; given severe vomiting and suspected bowel obstruction, the feasibility of oral administration was considered, and the patient was placed in airborne isolation, alongside total parenteral nutrition (TPN), vasopressor support, and broad-spectrum antibiotics. Despite aggressive multidisciplinary care, the patient’s condition deteriorated rapidly. He developed supraventricular tachycardia, electrolyte abnormalities, respiratory failure, and multi-organ dysfunction. Resuscitative efforts were unsuccessful, and the patient was pronounced deceased on November 16, 2025. This case highlights the devastating consequences of delayed diagnosis and management of extrapulmonary and miliary tuberculosis, particularly in the context of gastrointestinal involvement and malnutrition. It also underscores the impact of social determinants of health on disease progression and access to care in undocumented immigrant populations.

## Introduction

Tuberculosis (TB) remains a significant global health burden, particularly among socioeconomically disadvantaged and immigrant populations [1,2]. According to the WHO Global Tuberculosis Report 2022, TB remains a leading cause of infectious disease mortality worldwide, with extrapulmonary forms presenting major diagnostic and therapeutic challenges [3]. While pulmonary TB is well recognized, extrapulmonary manifestations such as gastrointestinal TB (GITB) are less common and remain diagnostically challenging due to their nonspecific clinical presentation [4]. GITB can present with vague symptoms such as abdominal pain, vomiting, and weight loss, frequently mimicking malignancy or inflammatory bowel disease [5]. Small bowel obstruction (SBO), a known complication of intestinal TB, can worsen prognosis by impairing nutritional intake and absorption [6].

Miliary TB, a severe form of disseminated TB, is characterized by hematogenous spread of Mycobacterium TB, resulting in numerous tiny nodules throughout the lungs and other organs, which is usually disseminated [7]. It can involve multiple systems simultaneously, making a timely diagnosis critical. Cachexia, defined as a multifactorial syndrome of severe weight loss, muscle wasting, and metabolic dysfunction, is a devastating sequela of chronic infectious diseases like TB [8]. Nutritional depletion not only exacerbates immunosuppression but also worsens clinical outcomes. When compounded by SBO and systemic infection, it creates a clinical scenario with high mortality risk [9].

## Case presentation

A 31-year-old male, who recently immigrated from India, presented to Chino Valley Medical Center on November 11, 2025, following a prolonged history of worsening symptoms. He reported chronic productive cough, nausea, severe fatigue, and an estimated weight loss of over 50 pounds in the preceding five months. He denied any prior history of TB, known TB exposure, chronic medical conditions, or substance use; his HIV status was negative, and his vaccination history was unknown. In the two weeks leading up to admission, he had been unable to tolerate oral intake and experienced near-constant vomiting. On arrival, he appeared emaciated and hypovolemic. His initial vital signs included a temperature of 98.3 °F, heart rate of 68 beats per minute, blood pressure of 103/73 mmHg, and oxygen saturation of 91% on room air.

Laboratory results on admission revealed critical findings: sodium was 128 mmol/L, hemoglobin was 7.0 g/dL, and serum albumin was as low as 1.3 g/dL, consistent with severe malnutrition and systemic illness [8]. Pertinent laboratory findings on admission are summarized in Table 1.

**Table 1:** Lab results upon admission.

Test	Result	Reference range	Interpretation
Sodium	128 mmol/L	135-145 mmol/L	Hyponatremia
Hemoglobin	7.0 g/dL	13.5-17.5 g/dL (male)	Severe anemia
Serum albumin	1.3 g/dL	3.4-5.4 g/dL	Severe hypoalbuminemia
Lactic acid	3.9 mmol/L	0.5–2.2 mmol/L	Lactic acidosis
Arterial pH (ABG)	7.469	7.35-7.45	Mild alkalosis
Oxygen saturation (SpO₂)	91%	95%-100%	Hypoxemia

**Figure 1:**
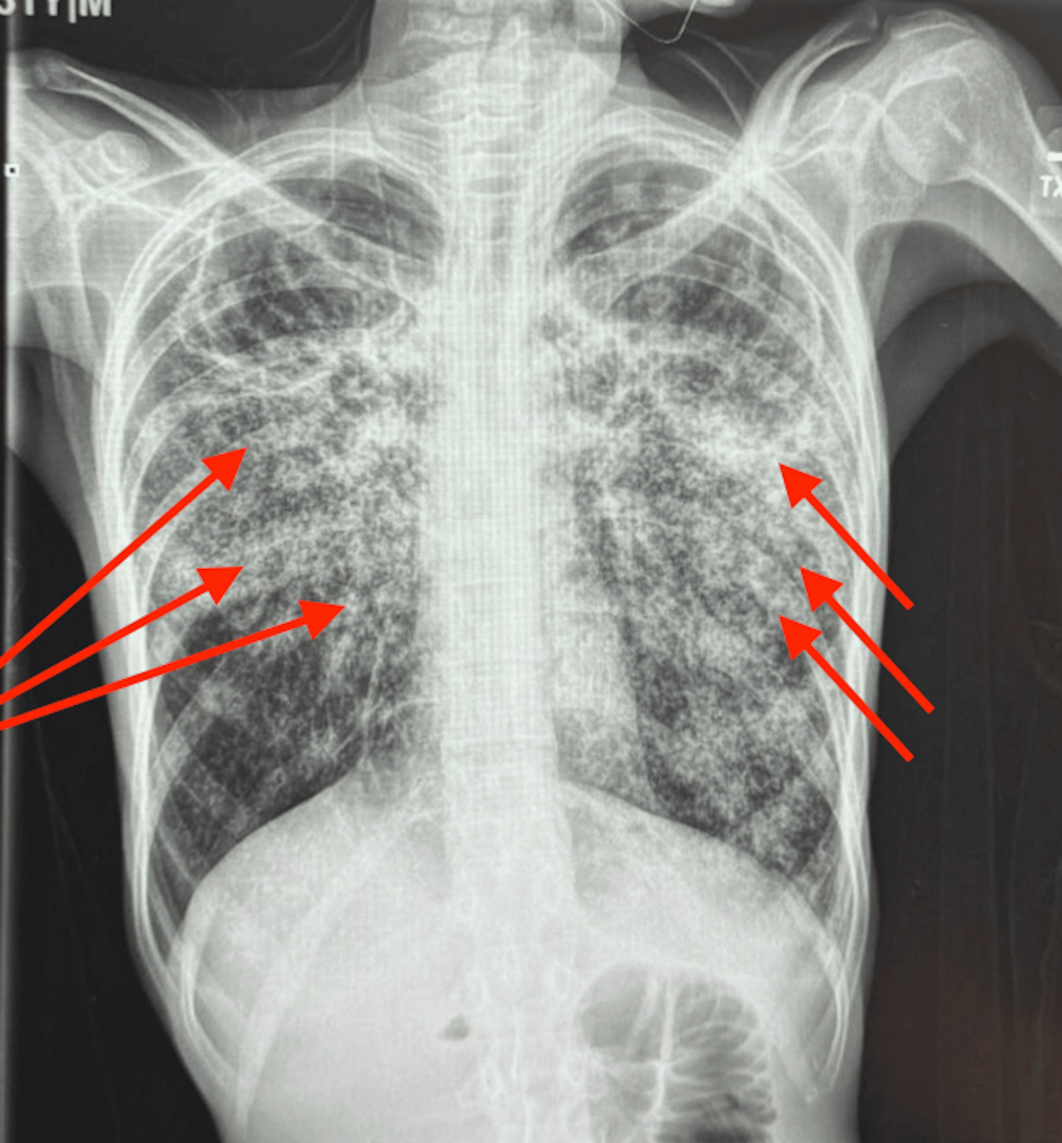
Chest X-ray (single view) showing bilateral reticulonodular infiltrates. Chest radiograph demonstrating diffuse bilateral reticulonodular and micronodular opacities throughout both lung fields (arrows), consistent with a miliary pattern of pulmonary disease.

Chest radiography performed on admission demonstrated bilateral reticulonodular infiltrates (Figure 1).

**Figure 2:**
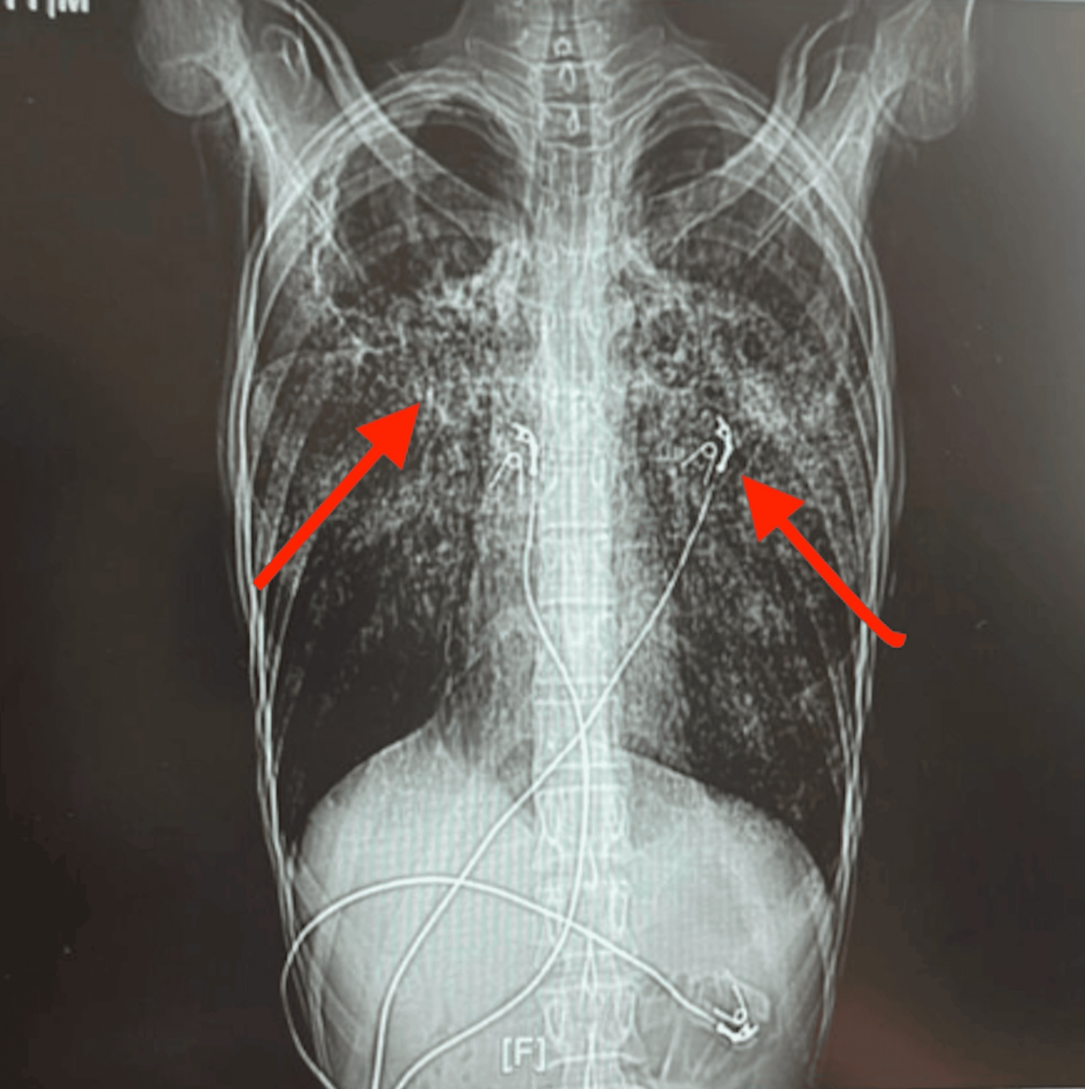
CT scan of the chest and abdomen without contrast. CT scan demonstrating diffuse bilateral micronodular opacities consistent with a miliary pattern of pulmonary tuberculosis (arrows).

A CT scan of the chest and abdomen revealed miliary nodules consistent with disseminated TB (Figure 2), which also demonstrated findings suggestive of SBO. Sputum culture later confirmed infection with Escherichia coli producing extended-spectrum beta-lactamase (ESBL).

By November 12, empiric anti-TB treatment was initiated with rifampin, isoniazid, pyrazinamide, and ethambutol, along with pyridoxine supplementation [10,11]. Due to persistent vomiting and intolerance to oral intake in the setting of suspected SBO, nasogastric decompression was initiated, and total parenteral nutrition (TPN) was subsequently started [12]. Surgical consultation advised against operative intervention for the SBO due to his fragile nutritional status and high perioperative risk [5]. Infectious disease, nephrology, gastroenterology, pulmonology, and cardiology services were all consulted for collaborative management.

On November 14, the patient's condition worsened. He became increasingly hypotensive, and he was treated with oral midodrine and intravenous vasopressor support with phenylephrine, later transitioned to norepinephrine. He developed supraventricular tachycardia and required transfer to the intensive care unit. Electrolyte abnormalities persisted, and serum sodium dropped further to 124 mmol/L. During clinical deterioration, empiric broad-spectrum antibiotic therapy with vancomycin and azithromycin was initiated for suspected infection while awaiting further microbiologic evaluation. Despite these measures, he showed signs of systemic decompensation, including worsening lactic acidosis and respiratory failure.

On the morning of November 16, the patient became bradycardic and hypotensive, and a code blue was called at 10:10 AM. Advanced cardiac life support was initiated, but resuscitation efforts were unsuccessful. He was pronounced dead at 10:23 AM on November 16, 2025, with presumed cause of death secondary to multiorgan failure in the setting of disseminated TB and severe sepsis. Written informed consent for clinical image documentation and publication was obtained from the patient before his passing in accordance with institutional policy.

## Discussion

This case demonstrates the severe clinical consequences of miliary and GITB in the setting of extreme malnutrition and delayed care [1]. GITB, although less common than pulmonary TB, can lead to complications such as small bowel obstruction due to granulomatous inflammation, strictures, and adhesions [4,5]. In this patient, abdominal imaging revealed bowel dilation and signs consistent with obstruction, further confirmed by clinical signs of vomiting and inability to tolerate oral intake [10].

The presence of miliary TB, indicated by the diffuse reticulonodular pattern on chest imaging and miliary nodules on CT, signifies hematogenous spread of *Mycobacterium tuberculosis*, placing the patient at high risk of multiorgan involvement [7]. Although RIPE therapy began on hospital day 2, the disease course suggests prolonged pre-hospital progression, the patient’s advanced disease and immunocompromised state, worsened by severe cachexia, limited the efficacy of treatment [8,9].

Cachexia in TB is driven by a proinflammatory state and metabolic derangements, leading to rapid wasting and hypoalbuminemia [8,13]. Studies confirm that even with nutritional support, reversal of severe cachexia in TB is difficult once advanced wasting sets in [13]. The patient’s serum albumin remained between 1.3 and 1.5 g/dL despite TPN, suggesting severe ongoing inflammation and poor nutritional reserve. Moreover, small bowel obstruction further hindered any potential for enteral nutritional recovery, removing the most physiologically favorable route of support [5].

This case also reflects the health disparities faced by undocumented immigrants. Barriers such as lack of insurance, delayed access to care, language differences, and fear of deportation may delay presentation until conditions become irreversible [2]. Several studies have reported higher TB morbidity and mortality among marginalized and migrant populations, often associated with delayed diagnosis, limited access to healthcare, and social barriers to care. Additionally, stigma and psychosocial stressors may further complicate healthcare access and treatment adherence in these populations [14].

Ultimately, the patient’s rapid deterioration, culminating in multi-organ failure, metabolic acidosis, and death, underscores the critical need for early recognition of extrapulmonary TB, timely nutritional intervention, and systems-level approaches to address social determinants of health [1,3,13].

## Conclusions

This case underscores the potential lethality of disseminated TB when diagnosis and treatment are delayed, particularly in socially vulnerable populations. It highlights the need for heightened clinical suspicion for miliary tuberculosis in patients with systemic illness and profound malnutrition. Early airborne isolation, expedited tuberculosis testing (including acid-fast bacilli (AFB) smear, culture, and nucleic acid amplification testing), and early involvement of infectious disease, nutritional support, and surgical evaluation for suspected bowel obstruction are critical for timely diagnosis and management. Additionally, the case draws attention to the impact of social and structural barriers on health outcomes, emphasizing the importance of accessible screening programs and equitable healthcare delivery to reduce preventable morbidity and mortality.
